# Comprehensive Biology and Genetics Compendium of Wilms Tumor Cell Lines with Different *WT1* Mutations

**DOI:** 10.3390/cancers13010060

**Published:** 2020-12-28

**Authors:** Brigitte Royer-Pokora, Maike Anna Busch, Sarah Tenbusch, Mathias Schmidt, Manfred Beier, Andrew D. Woods, Holger Thiele, Jaume Mora

**Affiliations:** 1 Institute of Human Genetics, Medical Faculty, Heinrich-Heine University, 40225 Düsseldorf, Germany; Maike.Busch@uk-essen.de (M.A.B.); sarah.tenbusch315@gmail.com (S.T.); Mathias.Schmidt@uk-essen.de (M.S.); Manfred.Beier@uni-duesseldorf.de (M.B.); 2 Children’s Cancer Therapy Development Institute, Beaverton, OR 97005, USA; andy@cc-tdi.org; 3 Cologne Center for Genomics (CCG), University Köln, 50931 Köln, Germany; hthiele@uni-koeln.de; 4 Department of Oncology, Hospital Sant Joan de Deu, 08950 Barcelona, Spain; Jmora@sjdhospitalbarcelona.org

**Keywords:** *WT1* mutant Wilms tumor cell lines, genetic and biochemical characterization of Wilms tumor cell lines

## Abstract

**Simple Summary:**

Wilms tumor is a childhood kidney tumor arising from embryonal cells. Wilms tumors are heterogeneous with several distinct subgroups that differ in their response to treatment. The genetic basis for these diverse forms of Wilms tumor is not fully understood. One subgroup of Wilms tumors is associated with mutations in the *WT1* gene, encoding a transcription factor with a role in early kidney differentiation. Patients with *WT1* mutant Wilms tumor may harbor germline mutations in this gene. Cell lines from Wilms tumors are notoriously difficult to establish and only few exist. We developed a method to cultivate cells from the *WT1* mutant subtype of Wilms tumors and have established 11 cell lines with different mutations in *WT1* to date. These cells will be instrumental to study the biology and genetics ultimately to develop precision treatments

**Abstract:**

Purpose: *WT1* mutant Wilms tumors represent a distinct subgroup, frequently associated with *CTNNB1* mutations. The genetic basis for the development of this subtype is currently not fully understood. Methods: Live *WT1* mutant Wilms tumors were collected during surgery of patients and cell cultures established in mesenchymal stem cell medium. They were studied for mutations in *WT1* and *CTNNB1*, their differentiation capacity and protein activation status. Four cell lines were immortalized with a triple mutant ts SV40 largeT antigen and Telomerase. Results: 11 cell lines were established from Wilms tumors of nine patients, including a left and right tumor from the same patient and a primary and second tumor from another patient. Six patients had germ line and three were tumor specific mutations. All cell lines harbored only mutant or deleted *WT1* genes. *CTNNB1* was wild type in three, all others carried mutations affecting amino acid S45. They had variable and limited capacities for mesenchymal differentiation, a high migratory capacity and a low invasive potential. All cells showed an activation of multiple receptor tyrosine kinases and downstream signaling pathways. Conclusions: These cell lines represent an important new tool to study *WT1* mutant Wilms tumors, potentially leading to new treatment approaches.

## 1. Introduction

The stromal subtype of Wilms tumor contains ectopic mesenchymal elements such as striated and smooth muscle cells, chondrocytes, osteocytes and adipocytes. This subtype of Wilms tumor often carries *WT1* mutations and most also harbor *CTNNB1* mutations [1,2,3]. These Wilms tumors represent an interesting biological group that needs further studies in order to better understand their biology and embryonal origin. Wilms tumor cell lines are not easily generated and various short-term cultures using defined serum free media have been described [4,5,6,7,8]. Several different culture media for Wilms tumor were tested but these did not help to establish long term cultures. At these early times, mutations in Wilms tumors were not known and therefore it was not possible to test whether the cultured cells were bona fide tumor or normal cells. After the identification of several recurrent mutations or LOH events in Wilms tumors the cultures could be analyzed for the presence of these mutations/alterations. Wegert et al., established short term cultures for 23 different types of Wilms tumors with known genetic alterations; only half of these had the same alterations as the tumor [9]. Two of these had mutations in *WT1* and in one case the *WT1* mutation was heterozygous, suggesting that the cells were not pure tumor cells [9]. One cell culture was described with a homozygous *WT1* mutation in the cultured cells that seem to express the WT1 protein. These cells showed senescence after passage 30 and were not characterized in detail [10]. We developed a new method to reproducibly establish long-term cultures of Wilms tumors with *WT1* mutations. Furthermore, these cell cultures can be easily transfected and transduced with lentiviral vectors. These cell lines are unique and will contribute to study *WT1* genetics in the context of Wilms tumor. 

Ten years ago, we described five cell lines with *WT1* mutations [11] and more recently we reported cell lines from a tumor and metastasis of the same patient; the tumor cell line was immortalized with ts SV40 largeT antigen (LT) and Telomerase [12]. Here we describe in detail the genetics, biological properties and molecular features of 11 *WT1* mutant Wilms tumor cell lines that we have established to date.

## 2. Materials and Methods

### 2.1. Patient and Tumor Characteristics

All cell cultures were initiated from fresh Wilms tumor samples obtained from patients between 1998 to date with a germ line or tumor specific *WT1* mutation. Patients 1 to 5 and patient 10 were described previously [11,12]. The tumors from patient Wilms1 and the genetic analysis of the bilateral first and second Wilms tumors has been described [13]. Patient 6 was diagnosed at 15 months with a bilateral WT and he had cryptorchidism. The cells were established from the left tumor. Patient 8 had a bilateral WT at 8 months, cryptorchidism and kidney cysts. The cells were established from the left tumor. Patient 11, a boy of 22 months had no genitourinary abnormalities and a predominant stromal WT in which large areas of rhabdomyomatous differentiation was observed. The tumor had invaded the ureter. He had a tumor specific mutation in *WT1* (Table 1). The study was approved by the local ethics committee (Nr. 2617) and parents gave written consent that leftover tumor material can be used for research. 

### 2.2. Cell Culture

Tumor cell culture was initiated by manual dissection of the tumor material with scissors into 1 mm cubicles, which were placed into the respective growth media, without disturbing for seven days. The detailed method has been described [11]. Cells were always kept at low density with a medium change every three days, they were sub cultured before they reached confluency. To study different tumor sections, we placed very small pieces of tumor material in individual wells of a 24 well plate. Once the cells in the wells were almost confluent, they were transferred to a 35 mm dish and thereafter to larger plates. Once a 35 mm plate was confluent, cells were frozen and DNA was isolated for further studies.

The cell cultures were tested for mycoplasma contamination and regularly for the presence of the specific mutations in *WT1* and *CTNNB1*. In addition, short tandem repeat validation was performed on all cell lines (Figure S1).

### 2.3. Mutation and LOH Analysis, Exome Sequencing and Varbank Analysis

All *WT1* exons and *CTNNB1* exon3 were analyzed for mutations by Sanger sequencing as described [3]. Blood, bulk tumor, microdissected and cell culture DNA was analyzed. For the detailed analysis of Wilms3, specific tumor areas from paraffin sections were identified. After deparaffinization, 3% peroxidase treatment and blocking, the slides were incubated with a Desmin antibody (DAKO, 1:200). Visualization of bound antibody was done with the Envison Plus-Peroxidase secondary antibody and DAB. The sections for manual dissection were circled and removed from the following slide at the marked position with a scalpel. DNA was extracted with the Acturus “PicoPure” kit containing Proteinase K at 65° for 20 h. Proteinase K was inactivated at 97 °C for 10 min. For the detection of specific mutations in the DNA from microdissected material, the respective exons were amplified and the PCR products were digested with enzymes recognizing only the mutant forms. The p.T41A *CTNNB1* mutation in exon3 of Wilms3 was cut with the enzyme *Bst*I and the Wilms3 p.V432fsX87 *WT1* mutation in exon 10 with *Mse*I. The products were analyzed on 2% agarose gels.

LOH analysis was performed on Wilms5 blood and tumor cell culture DNA using four microsatellite markers from chromosome 11p and 11q [11] and one CA-repeat marker from within the 3’UTR of *WT1* (BB6/7). The IRD800 labelled PCR products were separated on 6% polyacrylamide gels on a LICOR automatic sequencer. For whole-exome sequencing 1 μg of DNA was fragmented with sonication technology (Bioruptor, Diagenode, Liège, Belgium). The fragments were end-repaired and adaptor-ligated, including incorporation of sample index barcodes. After size selection, we subjected a pool of all 5 libraries to an enrichment process with the SureSelect Human All Exon V6 kit following manufacturer’s procedures (Agilent, Santa Clara, CA, USA).

The final libraries were sequenced on an Illumina HiSeq 4000 sequencing instrument (Illumina, San Diego, CA, USA) with a paired-end 2 × 75 bp protocol. The reads were then mapped to the Genome Reference Consortium human genome build 37 (UCSC version hg19) using the Burrows-Wheeler Alignment tool (BWA-aln) [14]. The 30× coverage was 88–95% of the target sequences. The Genome Analysis Toolkit (GATK) v.1.6 [15] was used to mark duplicated reads, perform local realignment around short insertions and deletions, recalibrate the base quality scores, and call SNVs and short indels. The deNovoGear software was used to detect de novo mutations [16]. Scripts developed inhouse at the Cologne Center for Genomics were applied to detect protein changes and affected donor and acceptor splice sites. The graphical user interface of the Varbank pipeline v.2.24 (https://varbank.ccg.uni-koeln.de//) was used to filter for de novo, high-quality, rare (MAF < 0.01) variants affecting protein structure or splice sites. To exclude pipeline related artifacts, the data were also filtered against an inhouse-database containing the exome sequencing data from 511 individuals with epilepsy.

### 2.4. Cell Migration and Invasiveness

Cell migration was analyzed and evaluated using the T-Scratch assay [17]. Cells were seeded in 35 mm dishes and at a confluence of about 80% a wound was scratched in the layer of cells with a 200 µL pipet tip. Medium was changed and fresh medium with or without serum was added. Photographs of the cells were taken at identical positions, immediately after the wound was placed and 4 and 8 h thereafter. The space filled in the wound with cells was determined with the T-Scratch software.

Invasiveness of the cell lines was studied using the CytoSelect™ Cell Migration Assay (Cell Biolabs, San Diego, CA, USA), consisting of a basement membrane-coated polycarbonate insert with an 8 μm pore size in a 24 well plate. The membrane serves as a barrier to discriminate migratory cells from non-migratory cells. Migratory cells that passed through the pores of the basement covered polycarbonate membrane were stained and quantified. 

### 2.5. Protein Extraction, Proteome Blot Analyses and Western Blot Analysis

For western blots, 20 µg of proteins were separated on small 10% SDS-PAGE and transferred to a PVDF membrane (BioRad Munic, Germany) P44/42 MAPK (Erk1/2) and phosphor-p44/42 MAPK (Erk1/2) antibodies (9102, 5726 respectively, Cell signaling, Leiden, The Netherlands) were used. The membranes were blocked with 5% milk powder in TBST and the antibodies were incubated in 5%BSA at a 1:2000 dilution for 2 h. A 1:2000 dilution of anti-rabbit IgG or anti mouse horseradish peroxidase linked (Cell Signaling, Leiden, The Netherlands) was incubated for 2 h and after washing, the bound antibody was detected using Amersham^TM^ ECL prime western blotting system (GE Healthcare, Uppsala, Sweden). The membrane was stripped and reprobed with an anti actin antibody. 

The following ProteomeProfiler^TM^ arrays (R&D Systems, Abingdon, UK) were used: for the parallel determination of the relative levels of the protein phosphorylation status of 49 tyrosine kinase receptors, the RTK antibody array kit (ARY001), for the phosphorylation of mitogen-activated protein kinases and other serine/threonine kinases, the MAPK antibody array kit (ARY002 and ARY002B). Antibodies against each phosphorylated protein are spotted in duplicates. Protein extracts were prepared as described in the protocol for the proteome profiler arrays (R&D Systems). The protein concentration was determined using the Bradford assay (BioRad). Two hundred µg of the respective proteins were incubated with the antibody arrays as described by the manufacturer (R&D Systems) and visualized with the Chemi Reagent mix supplied in the kit. 

### 2.6. Immunofluorescence Analysis

To analyze muscle differentiation with immunofluorescence, cells were seeded in four chamber slides (BD Biosciences, Heidelberg, Germany) and grown in reduced serum. The non-induced control cells were kept in MSC medium. The method to detect expression of Titin (TTN) after induction was performed as described [11]. 

Immunofluorescence staining for expression of cardiac TroponinT, a cTnT antibody (MA5-12960, ThermoFisher, Waltham, MA, USA) was used. Cells were fixed with 4% paraformaldehyde, washed 2× with 0.5 M glycine and 1× with PBS. For blocking, the slides were treated with 5% normal goat serum in PBS and 0.25% Triton X100 for 20 min. The cTnT antibody was used at a 1:200 dilution in PBS and 1% BSA and incubated for 1h at RT, washing was with PBS and 0.1% saponin. For visualization, the slides were treated with an Alexa Fluor 488 anti-mouse antibody at a 1:200 dilution in PBS with 1% BSA for 1h at RT. After washing, the chambers were removed and the slides were covered with Molecular Probes™ ProLong™ Gold Antifade Mountant (ThermoFisher) and a cover slip. 

For RAC1, CDC42, vimentin and cytokeratin immune staining, the cells were grown in 4 chamber slides (BD) in MSCGM (Lonza, Cologne, Germany). Fixation was performed with 2% paraformaldehyde and 4% sucrose in PBS for 15 min at room temperature, washed with PBS and blocked with 4% FCS and 0.1% Tween-20 in PBS for 30 min at room temperature. RAC1 staining was performed with rabbit polyclonal antibody at a 1:200 dilution (C-11; Santa Cruz Biotechnology, Dallas, TX, USA), CDC42 staining with a mouse monoclonal antibody diluted 1:200 (B9; Santa Cruz Biotechnology), Vimentin staining with a mouse monoclonal antibody at a 1:100 dilution (Mo725, Dako, now supplied by Agilent, Frankfurt, Germany) and Cytokeratin staining with a Pan-Cyto antibody at a 1:200 dilution (Sc 15367, Santa Cruz) in blocking solution at RT for 1 h. After PBS washing, they were incubated with goat-anti-rabbit AlexaFlour 546 or goat-anti-mouse AlexaFlour 546 secondary antibody (Invitrogen, now supplied by Thermo Fisher, Schwerte, Germany) for 1 h at room temperature in the dark. After final washing in PBS, cells were mounted in ProLong Gold Antifade with DAPI (Invitrogen). Immune fluorescence analysis of CDC42 and RAC1 was performed on a laser microscope (Zeiss, Jena, Germany). The microscope resolution for images was 60x for CDC42, and 40× for RAC1. 

Actin filaments were stained with fluorescent phalloidin. After washing of the cells with PBS they were fixed with 3.7% formaldehyde for 10 min, washed with PBS, followed by permeabilization with 0.1% Triton X-100 for 5 min, washed 3× with PBS and blocked with 1% BSA in PBS. Staining was performed with 1 Unit Alexa Fluor 555 Phalloidin (Invitrogen) in 1× PBS and 1% BSA for 20 min in the dark, followed by three washes with PBS. Cells were dried at RT and slides were covered with antifade mounting medium. 

### 2.7. Reporter Assays in WT Cells

For transfection of Wilms cells with the TOP- or FOPflash-reporter (firefly luciferase) plasmids (Upstate Biotechnology, now supplied by ThermoFisher, Ottawa, ON, Canada) cells were plated in six-well plates at a concentration of 1.5 × 10^5^ cells/well in MSCGM without antibiotics. At a confluence of 50–80%, the cells were cotransfected with TOP- or FOPflash-plasmids and as internal control for transfection efficiency with pRL-TK (Wilms1) or pRL-CMV (Wilms3) (Renilla luciferase) plasmids (Promega, Walldorf, Germany) using Lipofectamine LTX Reagent according to the protocol of the supplier (Invitrogen). All assays were done at least in duplicates or in triplicates and the firefly and Renilla luciferase activities were measured using the Dual-Luciferase Reporter Assay System (Promega) 24 h following transfection. The Renilla luciferase activity was used to normalize the transfection efficiency and the relative luciferase activity was used to calculate the TOP to FOP ratio.

Luciferase reporter containing four copies of the Smad binding site (SBE4-Luc; Addgene, Watertown, MA, USA) [18]) was used to test for the activity of the TGFβ pathway. Transfection was performed in triplicates and the standard error was determined.

### 2.8. Statistical Analyses

Statistical significance of fold-changes between sample replicates is based on p-values determined by pairwise *t*-tests (as implemented by the pairwise.t.test-function in R; https://www.R-project.org/) with pooled standard deviation, and Holm-adjusted for multiple testing. Due to the inherent log-normal distribution of expression data, all data was log-transformed prior to the analyses to meet the normality assumption of the *t*-test.

## 3. Results

### 3.1. Description of the 11 Cell Lines with Different WT1 Mutations

Cell cultures from 11 Wilms tumor samples were established in MSC medium from nine patients. First, we tested different media: (1) DMEM, (2) MSC and (3) the serum free medium described by Garvin et al., called here WT medium [5]. Stable long-term homogeneous cultures could only be established using MSC medium. Cell cultures from Wilms1 in DMEM resulted in cells that differentiated into multinucleated cells (Figure S2a) with shorter cultivation times. Furthermore, cells with the homozygous *WT1* mutation were gradually lost. Setting up cultures in WT medium resulted in outgrowth of normal epitheloid cells, that had only the germ line *WT1* mutation (Figure S2b).

The previously described Wilms1 cell line was derived from the second tumor of this patient from the left kidney (Wilms1-2l) and a new cell culture from the second tumor of the right kidney (Wilms1-2r), contained another mutation in *CTNNB1*, but the same homozygous *WT1* mutation. All mutations in *WT1* and *CTNNB1* found in the bulk tumor samples, in microdissected tumor samples, and derived cell lines are listed in Table 1. *WT1* mutations were homozygous in eight cell lines (Wilms1-2r+l, 2, 3, 5, 6, 8 and 11). The Wilms4 patient harbored a germ line 11p13 deletion and the established tumor cell line had a mutation in the remaining allele. A homozygous loss of *WT1* was present in Wilms10T and Wilms10M. Three of the nine patients (Wilms3, Wilms11, and Wilms10) had somatic *WT1* mutations. 

These *WT1* mutation analyses demonstrate that all cell lines had only mutant *WT1* genes and that they are bona fide tumor cells. This is further supported by immunofluorescence staining of the cell cultures with cytokeratin, that is negative in all and vimentin, that is positive in all WT cultures in all cells, i.e., these are homogeneous cultures of tumor cells. Examples are shown for Wilm1, 2, 6 and 8 in Figure S3.

We have shown previously that *WT1* mutant Wilms tumors display great heterogeneity of second *CTNNB1* mutations in vivo [19]. In the microdissected tumor from patient Wilms2, several different mutations in *CTNNB1* were detected [19]. In order to further analyze these finding we cultivated six explants from Wilms2 and these were sequenced for *CTNNB1* mutations. All six pools established from another vial of frozen cells had the same *CTNNB1* mutation (S45F) but different from the one we described for the first Wilms2 cell culture (S45Y). Importantly, both mutations were observed by microdissection of the original tumor (Table 1). This further confirms the heterogeneity of second mutations in *CTNNB1*. All WT-derived cell lines can be cultivated for at least 60 population doublings with a doubling time between 1.8 and 3 days. 

### 3.2. Karyotype, LOH, aCGH Analysis and Exome Sequencing of Wilms Cell Lines

A stable normal karyotype was observed in Wilms 1, 2, 4, 5, 6, 8 and 10, which was verified by aCGH in Wilms1, 2, 3 and 4. Wilms11 was not karyotyped. Wilms3 cells showed a mosaic karyotype at passage 16: 47,XY,+18 [19]/46,XY [4]. All Wilms cell lines had two normal appearing copies of chromosome 11 including the WAGR patient (Wilms4). LOH analysis showed that the cell line from patient Wilms4 retained both 11p parental alleles. This is supported by the higher expression of *H19* from the maternal allele in these cells than in all the other cell lines, harboring only paternal alleles (Figure S4). 11p11-pter LOH was shown in five of the cell lines (Wilms1-2r, 2, 3, 5 and 6). aSNP/aCGH showed that the Wilms10 LOH was limited to 11p15 [12].

Here we present data from exome sequencing of Wilms8. Sequencing was performed in the tumor DNA (Table S1) and the tumor cell culture DNA (Table S2) and compared to blood DNA. Eight tumor specific alterations were observed, one of these is not present in the tumor cell culture DNA (*FAM117B*). Four missense variants were heterozygous in the tumor sample and not present in blood (*FAM117B*, *CTNNB1*, *CELSR3* and *STXBP5L*). Two missense variants (*SAA2* and *SLC6A5*) were heterozygous in blood and homozygous in the tumor and both are located on chromosome 11 in the LOH region. The mutation in *WT1* is present in blood DNA and is homozygous in the tumor. A heterozygous stop mutation in *IGF2* is present in blood and it is homozygous in the tumor. This stop mutation is found in an N-terminally extended alternative *IGF2* transcript when compared to the canonical isoform1 and is described as a likely benign variant (rs200441006). Taken together, in this *WT1* mutant Wilms tumor no other clear pathogenic mutations except for *WT1* and *CTNNB1* are found. Sequencing of Wilms1, 6 and 10 will be presented in a forthcoming manuscript [20].

### 3.3. Wilms5: An Unusual Case

**Figure 1:**
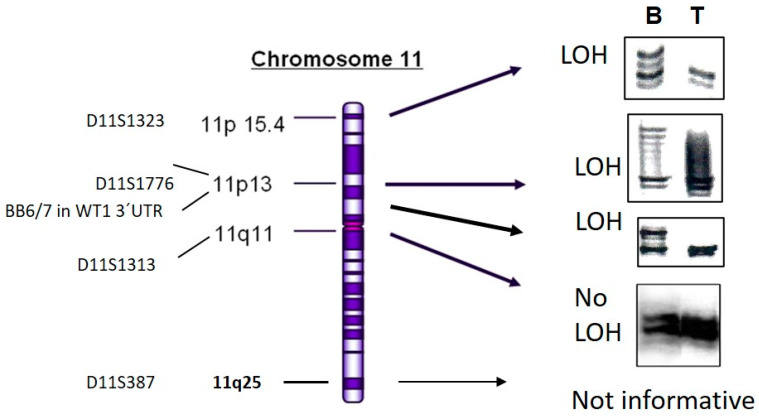
LOH analysis of DNA from patient Wilms5. DNA from tumor cell culture (T) and blood (B) of patient Wilms5 was analyzed with the indicated markers from chromosome 11. The IRD800 labeled PCR products were separated on 6% polyacrylamide gels. All markers from 11p15 to the 3´UTR of *WT1* show loss and the first marker more proximal in 11q11 shows no LOH. The marker from 11q25 was not informative.

The Wilms5 patient had a germ line *WT1* p.R390X mutation. Tumor DNA was heterozygous for p.R390X and wild type for exon3 and exon8 of *CTNNB1*. The cell line was established from a fresh tumor sample obtained in medium. Surprisingly at passage 2 in culture the germ line p.R390X *WT1* mutation was not detected. To exclude contamination or mix up of cells, several CA repeat markers were tested resulting in identical patterns for blood and cell culture DNA. To further analyze this unusual result, the entire *WT1* gene was sequenced and uncovered a different *WT1* mutation: a homozygous deletion of 2 nucleotides in exon10, p.R433PfsX84. After reinspection of the tumor DNA sequence a very low profile of a mixed sequence starting at the point of the deletion in exon 10 was observed. This suggests that a low number of cells with the exon 10 deletion was present in the original frozen tumor sample. Sequencing revealed a C/T polymorphism in intron 9 in blood DNA and the tumor sample, whereas the Wilms5 cell line without the p.R390X mutation was homozygous for the C allele. This indicates that the *WT1* germ line mutation p.R390X was present on the T allele. CA repeat marker analysis identified loss of markers distal and retention of markers proximal of 11p11 (Figure 1). This reflects a mitotic recombination occurring between 11p11 and 11p13. Taken together, we can conclude that the cells in culture were derived from a cell clone that has lost the mutant p.R390X allele, followed by acquisition of a new mutation in *WT1*. The resulting homozygosity of the new mutant allele might be due to another mitotic recombination event. The cytogenetic analysis showed two copies of chromosome 11. Interestingly, the tumor from the patient was later described by pathology as an intralobar nephroblastomatosis (ILNR), not Wilms tumor. In agreement with pathological classification as ILNR, the established cells had no mutation in *CTNNB1*.

### 3.4. Analysis of Cell Lines without CTNNB1 Mutations

**Figure 2:**
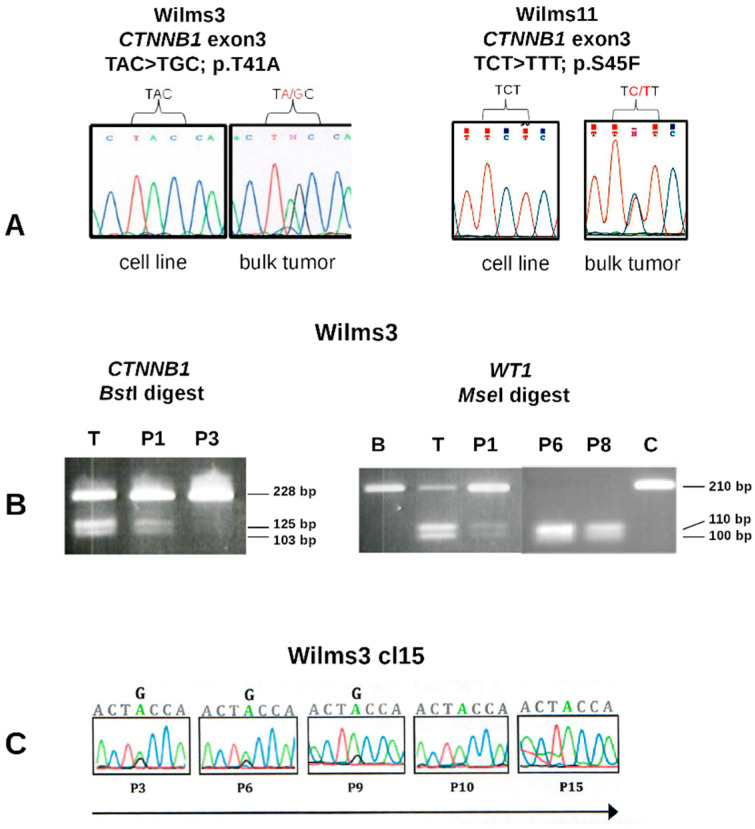
*WT1* and *CTNNB1* sequence analysis Wilms3 and Wilms11 DNA. (**A**) Partial sequence of *CTNNB1* exon3 from cell lines and bulk tumor DNA from patient Wilms3 (left) and Wilms11 (right) with the mutations indicated. (**B**) Restriction enzyme digest of PCR products with *Bst*I, recognizing only the mutant *CTNNB1* exon3 DNA (left) and *Mse*I recognizing only the mutant *WT1* exon10 (right). T: bulk tumor DNA; B: blood DNA, P1, P3, P6 and P8 correspond to DNA isolated from cell culture in MSC medium at the respective passage number, C = control DNA. (**C**) DNA isolated from Wilms3 cl15 cells was sequenced and the *CTNNB1* mutation is gradually lost and at passage 10 not present anymore.

In two of the three patients with somatic *WT1* mutations, Wilms3 and Wilms11, no mutations in *CTNNB1* were observed in the cell lines in comparison to the tumor DNA carrying a p.T41A and a p.S45F *CTNNB1* mutation, respectively (Figure 2A). The Wilms3 cell line lost the *CTNNB1* mutation early during culturing in MSC medium, as it was seen in passage 2 but was absent at passage 3 (Figure 2B, left panel). In Wilms3 bulk tumor DNA also small amounts of wild type *WT1* was observed, whereas a homozygous *WT1* mutation was seen in cells cultured in MSC medium at passage 6 (Figure 2B, right panel).

To test whether the *CTNNB1* mutation seen in the original tumor was observed in specific cell types, DNA of two different tumor sections were microdissected after desmin staining (Figure S5). DNA from the following areas were isolated: from slide1, two different areas containing some unstained cells and many desmin positive rhabdomyoblastic cells (1.1 and 1.2), one area containing mostly stroma/interstitial tissues and few desmin positive cells (1.3) and one with few tubuli (1.4). A normal appearing kidney section of the same slide was used as control (1.5). From slide 2, similar areas were identified and additionally DNA from an area that contained blastema was isolated (2.4) (Figure S5). The extracted DNA could not be sequenced and therefore, the mutation was identified by restriction enzyme digestion of PCR products. Figure S6 shows that only one tumor section with mainly desmin positive cells had a homozygous *WT1* mutation (Figure S6A, lane 2). All other dissected areas contained mutant and wild type *WT1* and the normal section is wild type for *WT1* (Figure S6A, lane5). Figure S6B shows that all sections except for the normal kidney (lane 5) contain mutant and wild type *CTNNB1* in variable concentrations.

In order to study these further, small tumor explants were cultured in 24 well plates and were individually passaged. These cultures were derived from a mix of tumor cells; thus, they are pools of cells and for simplicity they were called clones. 14 of 24 clones sequenced carried a mutation, most at a lower level than wild type and only three cultures had a 1:1 ratio of mutant and wild type *CTNNB1* alleles. Clone 15, with a 1:1 ratio was passaged further in MSC medium and the *CTNNB1* mutation was gradually lost and could not be observed after passage 10 (Figure 2C). Taken together, if Wilms3 cells with a homozygous *WT1* and a *CTNNB1* mutation exist, they are not able to grow in MSC medium.

In the case of Wilms11, three tumor explants were cultured separately and all three were wild type for *CTNNB1* (Figure 2A, right). This result confirms what we had observed for Wilms3, that tumors arising in a patient with a somatic *WT1* mutation, might have cells in the tumor with *WT1* (possibly heterozygous) and *CTNNB1* mutations, but these do not grow in MSC medium.

### 3.5. Comparison of Wilms3 Cells with and without CTNNB1 Mutations

**Figure 3:**
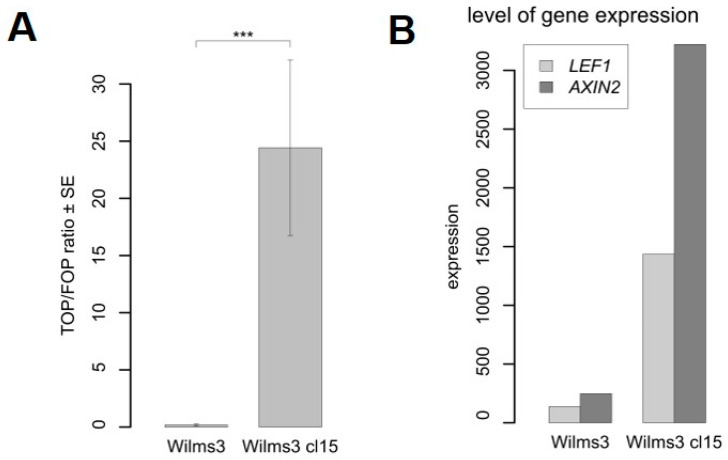
Analysis of the Wnt signaling pathway in Wilms3 cells with or without mutant *CTNNB1*. (**A**) Transient transfection assay of Wilms3cl15, with *CTNNB1* mutation and Wilms3 cells with wild type *CTNNB1*, with TOP- and FOP-flash reporter plasmids and analysis of the luciferase activity. The TOP to FOP ratio is displayed relative to the internal *Renilla* control plasmid. The standard error was derived from three independent experiments. *** = *p*-Value < 0.001. (**B**) Level of Wnt target gene expression in Wilms3cl15 and Wilms3 cells determined by Agilent whole genome expression arrays.

The Wilms3 cl15 with a mutant *CTNNB1* and the original Wilms3 cells were tested for an active Wnt signaling pathway using transfection with TOP/FOP reporter gene constructs. The ratio was determined after normalization for transfection efficiency with a *Renilla* reporter construct. Figure 3A shows that the TOP luciferase activity is significantly higher (*p* = 0.0002) in Wilms3cl15 cells with a mutant *CTNNB1* compared to Wilms3. This is corroborated by the higher expression level of the Wnt target genes *AXIN2* and *LEF1* in the mutant cells (Figure 3B).

### 3.6. Immortalization

The primary Wilms tumor cell lines have a limited life span, similar to MSC cells. Therefore, we immortalized four cell lines (Wilms1, Wilms3, Wilms6 and Wilms10) with triple mutant (U19dl89-97tsA58) SV40 large T antigen (LT). Immortalization with this ts LT results in chromosomally stable cell lines [12]. In parallel MSC cells were also immortalized. As LT alone does not result in immortalization of many cell types, *Telomerase* (h*TERT*) was used in addition. The immortalized Wilms10 cells (imWilms10) have been described [12]. The cells with the ts LT are cultured at the permissive temperature (33 °C) and a switch to the nonpermissive temperature (37 °C) leads to inactivation of the large T antigen. Thus, the cells can be tested in a more native state. 

Immortalized MSC grow well at 33 °C and a switch to 37 °C leads to a gradual growth arrest after a few passages. Surprisingly, the four Wilms tumor cell lines continued to grow normally at 37 °C for at least 100 passages, corresponding to 300 population doublings. The imWilms1 and imWilms10 cells had a stable normal karyotype at all tested passages, imWilms6 was not analyzed and imWilms3 had a gain of chromosome 7 and the stable karyotype was 48,XY,+7,+18 [13]. 

### 3.7. Analysis of Differentiation Potential of the WT1 Mutant Wilms Tumor Cell Lines

The differentiation potential of Wilms1, 2, 3, 4, 5 and 10 has been described [11,12]. Here, we describe the muscle differentiation potential of Wilms6, 8 and 11 using an antibody for Titin (TTN) after growth in serum reduced medium as described [11]. In Wilms6 and 8 most cells were TTN positive after muscle induction and some of these were multinucleated (Figure S7). In addition, Wilms10 and 11 cells were tested for cardiomyocyte differentiation using a cardiac troponin T (cTnT) antibody; an example for Wilms10 is shown in Figure S7, bottom. Nine Wilms10 cells were positive before and 140 after induction in the view field (Figure S8). In Wilms11, 4.5% of the cells were cTnT positive before induction and 29% after differentiation induction (Figure S8). In summary, *WT1* mutant Wilms tumor cell lines have a limited and variable mesenchymal differentiation capacity.

The immortalized Wilms1, and 3 cells did not show adipogenic or osteogenic differentiation potential. The immortalized Wilms10 cells were shown to have a reduced differentiation potential for the osteogenic and adipogenic lineage when compared to parental nonimmortalized cells [12]. In addition, they have a similar muscle differentiation potential as the parental non-immortalized cells [12]. ImWilms6 were not tested for any differentiation potential.

### 3.8. Migration and Invasiveness

**Figure 4:**
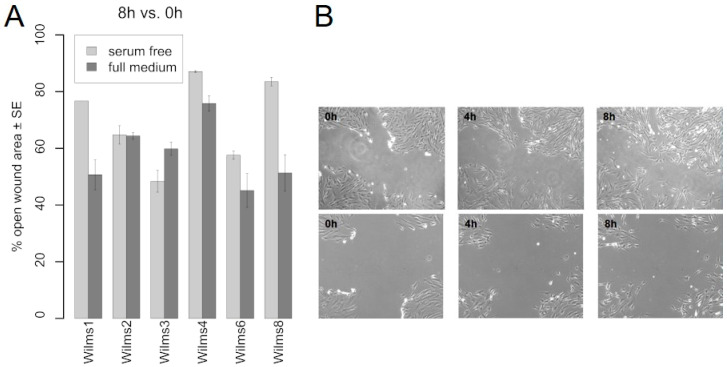
Migration properties of Wilms cell lines. (**A**) Migration of the indicated Wilms cell lines into the wound in serum free and full medium is shown. The percentage of open wound left after 8 h is indicated. (**B**) Example of Wilms8 cell growth in the wound in full medium, top panel and serum free medium, lower panel. Magnification 20×.

The migration capacity was tested for Wilms1, 2, 3, 4, 6 and 8 cells in serum free and serum containing medium using the T-scratch assay. Figure 4A shows the results as determined by the percent of the open wound that the cells occupy after 8 h. Wilms2 cells have the same migration capacities in serum free and full medium and Wilms3 cells seem to migrate even faster without serum. All other cells migrate less in serum free medium. An example of Wilms8 cell growth into the wound is shown in Figure 4B. 

Focal complexes are formed at the leading edge of migrating cells and this depends on RAC1 and CDC42. Wilms6 cells were analyzed for the expression and localization of CDC42 and RAC1. CDC42 shows perinuclear staining and several dot-like structures at the cell surface (Figure S9). RAC1 stains several broad lamellipodia like clusters, suggesting that the cells might have more than one leading edge (Figure S9). In addition, Wilms6 cells have a less ordered actin filament typical for tumor cells when displayed by phalloidin-rhodamine staining (Figure S10).

**Figure 5:**
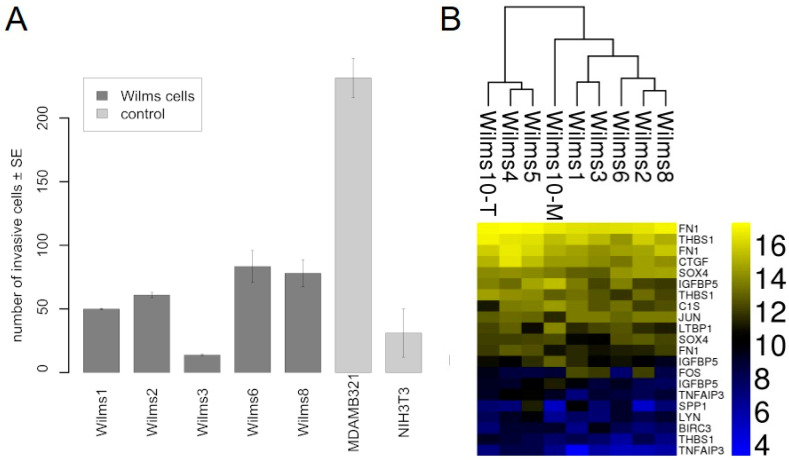
Invasion properties of Wilms cell lines. (**A**) The number of cells that invaded the basement membrane is shown for the tested Wilms cell lines. As controls we used nonmalignant NIH3T3 fibroblasts and MDAMB321, a highly invasive breast cancer cell line. The experiment was repeated twice and the standard error is shown. (**B**) Heat map of the CIG cluster expressed in all Wilms cell lines.

The invasiveness of Wilms1, 2, 3, 6 and 8 cells were tested by their potential to degrade and pass through a matrix covered polycarbonate membrane, towards serum containing medium. Figure 5A shows that all cells have a low invasive potential, but clearly less than the MDAMB321 breast cancer cells. Wilms3 was less invasive than non-malignant NIH3T3 cells, i.e., they are noninvasive.

Additionally, the core invasiveness gene cluster (CIG) identified by (Marsan et al., 2014) consisting 16 genes was studied in Wilms cell lines [21]. The heat map in Figure 5B shows that 13 of these genes are expressed at various levels in all Wilms cell lines. The highest level in all cell lines was observed for *FN1*, *THBS1* and *CTGF*.

### 3.9. Protein Activation Status of the WT1 Mutant Wilms Tumor Cells Lines

**Figure 6:**
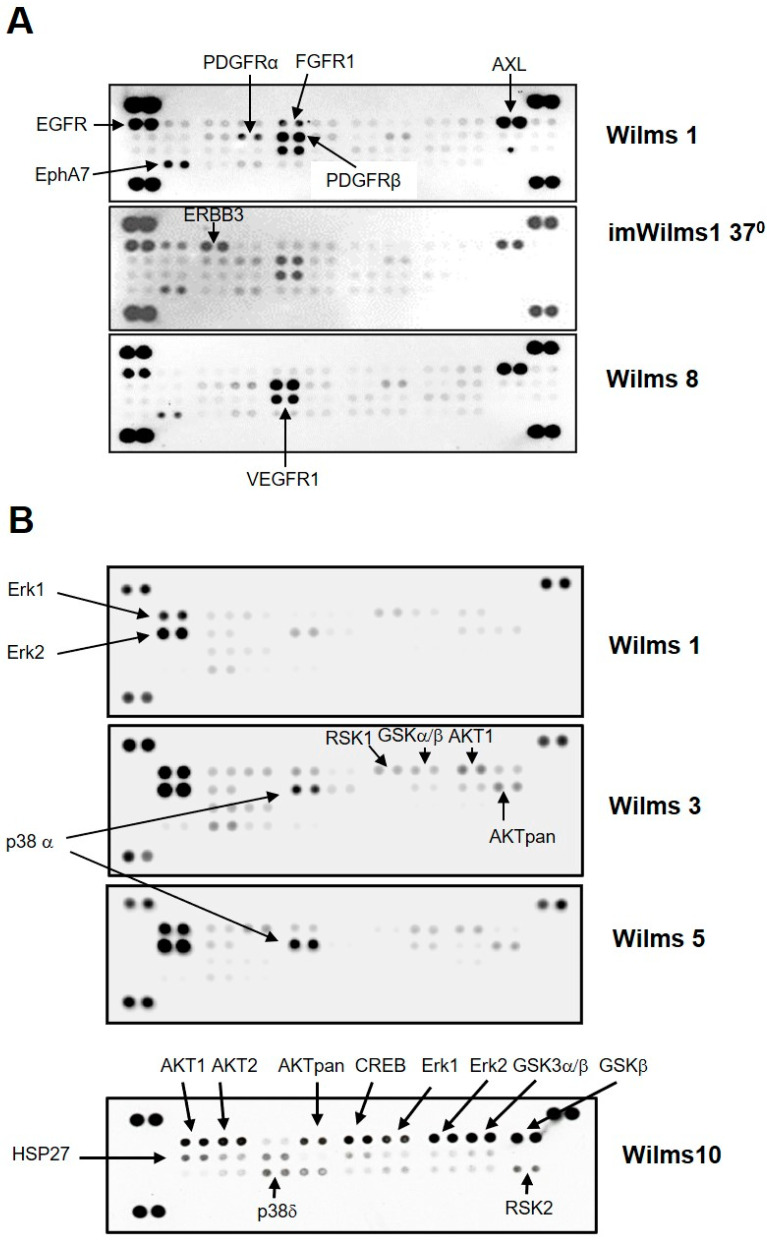

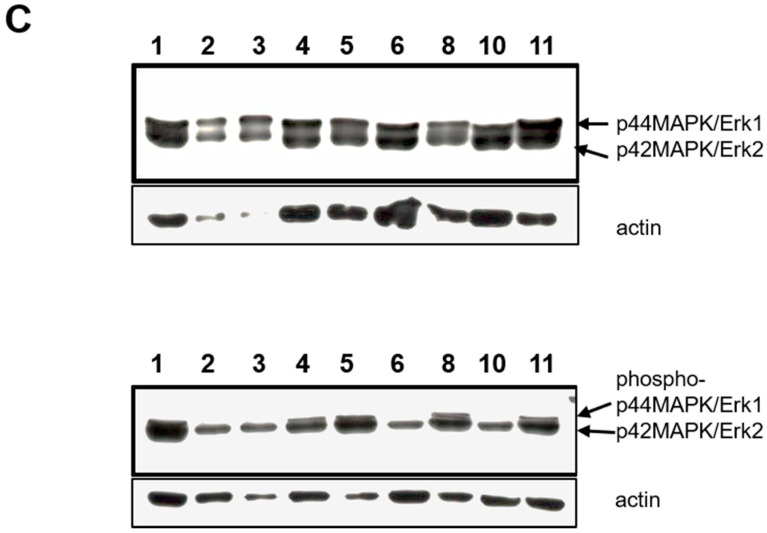
Phosphorylation of Receptor tyrosine kinases and MAP kinases in Wilms cells. (**A**) Human Phospho-RTK array. Each RTK is spotted in duplicates. Representative examples of results for Wilms1, imWilms1 cultured at 37 °C and Wilms8 are shown. The intensity of the spots corresponds to the level of phosphorylation of the proteins. The strongest phosphorylated receptors are highlighted with an arrow and their names are listed. For example, FGFR1 phosphorylation is stronger in nonimmortalized Wilms1 cells, and ERBB3 is more phosphorylated in imWilms1 cells. All other Wilms cells have similar phosphorylation patterns. (**B**) Human Phospho-MAPK array. Examples for Wilms1, Wilms3 and Wilms5 are shown (top part of panel). Later the design of the array format and order of spotted MAPKs changed, an example of this newer format is shown for Wilms10. Some of the proteins are not represented on the older format (top panel). (**C**) Western blot of protein extracts from the Wilms tumor cell lines and probed with pErk1/2 (top) and phospho-pErk/2 antibodies. The number on the lanes corresponds to the extracts from the respective Wilms cell lines. The same blot was reprobed with an actin antibody as loading control. The original film is shown in Figure S12.

To test which receptors or downstream signaling pathways are active in the Wilms tumor cells, we used proteome profiler arrays. The first analysis was done to study the general phosphorylation status of 48 receptor tyrosine kinases (RTK), corresponding to their active state. Using protein extracts from WT cell lines we identified the activation of several receptors. Figure 6A shows an example of the results for Wilms1, imWilms1 and Wilms8 cells. The results were similar for all the other cell lines. The strongest phosphorylation was observed for EGFR, PDGFRβ and AXL. Less phosphorylation was observed for PDGFRα, EPHA7 and VEGFR1. ImWilms1 cells showed additional phosphorylation of ERBB3, whereas the non-immortalized cells had more active FGFR1. In addition, Wilms10 cells also had an activated IGF1R [12].

Downstream signaling was analyzed using the phosphorylation status of MAP kinases. An example of the results for Wilms1, 3, 5 and 10 is shown in Figure 6B. The most highly phosphorylated kinases were Erk1 and Erk2. In Wilms3 and Wilms5 cells with wild type β-Catenin, phosphorylation of p38α was also seen along with a weak phosphorylation of AKT1 and AKTpan as well as activation of CREB, only present on the newer array format. The phosphorylation of GSK3α/β corresponding to the inactive state, associated with activation of the Wnt signaling pathway was observed in Wilms10, (Figure 6B). The phosphorylation of Erk1/2 was confirmed by western blots. Both proteins were present in about equal amounts (Figure 6C, top panel), but Erk2 shows a stronger phosphorylation (Figure 6C, bottom panel), confirming the results of the proteome blots. 

Our previous gene expression studies indicated an active TGFβ signaling cascade and therefore, TGFβ pathway activation was tested by transfection of a Smad target reporter construct (SB4E) in Wilms1 and imWilms1 cells. This assay showed an activation of the canonical TGFRβ/Smad pathway (Figure S11). Using a gene set of 188 genes that was induced by TGFβ in NCLC [22] we tested how many of these genes are expressed in all Wilms cell lines. As a cut off, an expression intensity of 500 (Agilent array) was used and the heat map shows a robust expression of 82 of these in all Wilms cell lines (Figure S13). In summary, multiple receptor tyrosine kinases are phosphorylated/activated and several downstream oncogenic signaling pathways are active in Wilms tumor cell lines.

## 4. Discussion

To explore the molecular basis of the *WT1* mutant subgroup of Wilms tumors, in vitro culture models are highly desirable. Our initial motivation to use MSC medium to establish cell cultures from these tumors was that in *WT1* mutant tumors a mesenchymal differentiation pattern is often observed. Here we show that long term cell cultures from these tumors can reproducibly be established in this medium, even from patients with preoperative chemotherapy. In this work we present an overview of the biological and genetic properties of 11 *WT1* mutant cell lines. Our previous description of Wilms1-5 cells demonstrated that they are similar to MSC cells and express the stem cell surface markers CD105, CD90 and CD73 [11]. The gene expression analysis showed that a number of the genes were specifically expressed in the Wilms tumors when compared to MSC cells [11]. Here, we add more information and compile a comprehensive summary of their biological and genetic properties, as well as their proteome activation status. 

All Wilms cell lines presented here can be grown for at least 60 population doublings in culture, irrespective of the presence or absence of an activated mutant β-Catenin but they are not immortal. All cells have a stable normal karyotype, and Wilms3 had mosaic trisomy 18. Importantly, imWilms1 and imWilms10 cell lines also had a stable normal karyotype during all passages. ImWilms3 cells showed a gain of chromosome 7 in addition to the gain of chromosome 18, but this did not change during culturing. The immortalized cells can be cultured indefinitely at 37 °C, although they were immortalized with a triple mutant ts LT. Immortalization of the Wilms cell lines with h*TERT* alone was not sufficient, indicating that for the establishment LT is necessary. 

The different *WT1* mutations were homozygous in eight cell lines, in five that were analyzed for LOH, this was due to a mitotic recombination event proximal of the *WT1* gene, therefore all genes on the short arm of chromosome 11 showed uniparental disomy (UPD). In one case the parental origin was shown to be paternal UPD, supported by the high expression level of *IGF2* from the paternal allele in 11p15 and the almost absent maternal expression of *H19*. In the WAGR cell line (Wilms4) the remaining allele carried a *WT1* mutation and there was no UPD in 11p. A homozygous deletion was present in Wilms10T and Wilms10M and SNP analysis revealed that UPD was limited to 11p15 but containing the *IGF2* gene [12]. The cell line Wilms5 did not harbor the germ line mutation but a different homozygous *WT1* mutation and *CTNNB1* was wild type. The lack of a *CTNNB1* mutation in this cell line established from an ILNR confirms previous observations that ILNRs do not harbor *CTNNB1* mutations [23]. The presence of only mutant *WT1* alleles in all cell cultures confirms their tumor origin and the homogeneity of the cultures.

In all Wilms tumor cell cultures with a *CTNNB1* mutation serine 45 is affected. S45 is a functionally important amino acid in the protein as it is the site of phosphorylation by serine-threonine kinase GSK3β. Phosphorylation at several sites in the N-terminus of β-Catenin is crucial for degradation by the ubiquitin proteasomal complex. Mutations affecting this amino acid result in stabilization, nuclear accumulation and constitutive activation of the Wnt pathway. Serine 45 was changed to phenylalanine (F), cysteine (C) and tyrosine (Y); a deletion of serine 45 (∆S45) was observed in three cell lines. Of note, in three cases the ∆S45 was homozygous (Wilms6, Wilms10T, Wilms10M); in Wilms10 this is due to UPD 3p [12]. A homozygous *CTNNB1* mutation is not normally observed in tumor cells as the mutant allele functions as a dominant oncogene. It would be interesting to study whether the different mutant β−Catenin proteins have variable functional impacts in the Wilms cells. 

Two cell lines (Wilms3 and Wilms11) were established from tumors where the bulk tumor had a *CTNNB1* mutation but the cell cultures were wild type. In Wilms3 the tumor carried a p.T41A *CTNNB1*, an amino acid that is also a target for phosphorylation by GSK3β, and the Wilms11 tumor carried a p.S45F mutation. In both cases the *WT1* mutation was tumor specific, i.e., not present in the germ line. Further studies of Wilms3 showed that cells with a *CTNNB1* mutation did not survive in the MSC medium. This indicates that the tumor cell cultures first consist of a mix of cells and those cells harboring a *CTNNB1* mutation were lost in culture. Experiments to set up Wilms3 cells in WT medium [5] showed, that cells with a more epithelial appearance grow out in this medium. These were wild type for *WT1* and *CTNNB*, i.e., were normal kidney cells [11]. Cells with a heterozygous *WT1* mutation (first hit) that also harbor a *CTNNB1* mutation could not be identified in our cell culture conditions but they may exist in the tumor in vivo. Once a homozygous *WT1* mutation is present, the cells may be independent for an additional *CTNNB1* mutation and could be the seeds for tumor development. In a sporadic *WT1* mutation case the first mutation occurs in a specific kidney cell, likely followed by a *CTNNB1* mutation in the same cell, but this cell might not form a tumor. If the *WT1* mutation becomes homozygous and if it acts as a gain of function, these cells do not necessarily need a *CTNNB1* mutation. In this context it is important that we have shown previously that the WT1^Wilms3^ mutant protein is expressed and it regulates a set of cell cycle genes [24]. The mutant protein with a normal N-terminus has retained its ability to bind to RNA and some transcription regulation function but has lost its DNA binding activity. Consequently, the normal *WT1* target genes are not regulated, but the mutant gene has gained new functions and is therefore a gain of function mutation [24]. The fact that these cells are wild type for *CTNNB1* suggest that this gain of function mutation in *WT1* has an oncogenic function independent of β-catenin. 

Here we show that all Wilms cell lines have a limited mesenchymal differentiation potential similar to MSC. Furthermore, after muscle differentiation induction, they also express markers of cardiomyocytes. The cells express spontaneously, i.e., without differentiation induction several marker genes that are specific for adipocytes, chondrocytes or osteocytes [25]. The expression levels vary between cell lines, confirming their intrinsic but diverse potential for differentiation. The level of the marker gene expression might be dependent on the culture conditions and their partial spontaneous differentiation. 

Cell migration is a multistep process involved in many normal physiological processes such as embryonic morphogenesis, tissue repair and regeneration. Its aberrant regulation drives cancer invasion and metastasis. Cell migration is initiated by polarization and protrusions that are termed lamellipodia or spike-like filopodia [26]. These structures are formed by actin polymerization at the leading edge of the migratory cells. Staining of the Wilms tumor cells with a RAC1 antibody revealed that lamellipodia can be observed at several sites on the cell and not only in the direction of movement. Additionally, they have a less well-organized actin filament that resembles those in malignant cells. All Wilms cells have a high migratory activity and two of the cell lines (Wilms2 and 3) show serum independent migration and both cell lines express a mutant WT1 protein that can act as an oncogene [21]. Another important observation was that Wilms6 and 8 with the same *WT1* mutation but different *CTNNB1* mutations differ in the migration property. Wilms6 migrates more efficiently in the absence of serum and has a homozygous p.∆S45 β-Catenin mutant protein. Wilms8, where migration is serum dependent has a heterozygous p.S45A mutation. This indicates that the type of *CTNNB1* mutation contributes to their migration property.

The ability of malignant tumor cells to invade normal surrounding tissue contributes to their metastatic potential. Invasiveness requires several cellular functions including adhesion, motility, detachment, and extracellular matrix proteolysis. Metastatic cells produce many proteolytic enzymes (e.g., lysosomal hydrolysates, collagenases, plasminogen activators) while the expression of certain cell surface protease receptors is also increased. Invasiveness is initiated by an enzymatic degradation of the barrier. Recently, a core invasiveness gene cluster (CIG) has been described [21]. Thirteen of these 16 CIG genes are expressed at high levels in all WT cell lines. Five Wilms tumor cell lines were tested for their ability to degrade and invade through a synthetic ECM matrix. A low invasive potential was observed for Wilms1, 2, 6 and 8. Although Wilms3 cells did not show an invasive potential, they express the same high level of the core invasiveness genes as the other cell lines. 

All Wilms tumor cell lines have a major activation of three tyrosine kinase receptors: EGFR, PDGFR and AXL, detected by their strong phosphorylation. EGFR signaling plays a role in proliferation, migration, growth and differentiation. EGFR family members are crucial for nephrogenesis and are expressed in glomeruli, tubules and the interstitium at early and late gestational stages [27]. Downstream signaling cascades includes the MAPK pathway, depending on Ras-Raf-MEK and Erk activity and PI3K signaling depending on AKT kinase. The Erk/MAPK signaling is closely related to cell proliferation and differentiation and is frequently dysfunctional in cancer [28]. The most significant phosphorylation detected in all the Wilms cell lines was Erk1/2. In addition, a lower level of AKT phosphorylation was also found. A high level of Erk1/2 phosphorylation was also observed in the *Wt1-Igf2* Wilms tumor mouse model and was postulated to be due to Igf2 signaling through IGF1R activation [29]. In the human *WT1* mutant Wilms tumor cells we did not observe a phosphorylation of this receptor in the proteome studies, although *IGF2* RNA expression was high. This suggests that in the human cells the Erk/MAPK pathway activation occurs though another phosphorylated receptor.

The activation of PDGFR induces specific sets of genes that determine fate and behavior of different cell types [30] and one of the major functions of PDGF is the regulation of cell migration [31]. Knockout mice uncovered that PDGFRβ signaling is necessary for recruitment of mesangial cells to the glomerulus [27]. Kidney interstitial mesenchyme expresses PDGFRα and its formation occurs through the combined activity of the ligands PDGFA and PDGFC [31], both are expressed at the RNA level in Wilms cell lines. PDGFR dimerization results in autophosphorylation and activation of the kinase that provides docking sites for downstream signaling molecules. Both receptors engage several signaling pathways, similar to EGFR activation. The growth and survival of the cells may depend on receptor crosstalk and heterodimerization, i.e., treatment of COS7 cells with EGF induces rapid phosphorylation of PDGFRβ [32]. In addition, transactivation of EGFR by PDGF stimulation is required for PDGF mediated cell migration [33]. We found evidence for possible heterodimer formation between EGFR and PDGFR by co-down regulation of their phosphorylation after inhibition of EGFR with Erlotinib (Busch and Royer-Pokora, unpublished results). Moreover, although the RNA of the ligands for EGFR are expressed at very low levels, a high phosphorylation of EGFR is observed. The RNA for the ligands for PDGFRα/β, *PDGFA* and *PDGFC* are expressed at high levels and these two receptors show less phosphorylation. In addition, the RNA for the AXL ligand, *GAS6* is also expressed at very high levels in all Wilms cell lines. Therefore, autoregulation and transactivation of these receptors may be active in the Wilms cells.

We also identified the activation of canonical TGFβ signaling via Smad4 in Wilms1 and imWilms1 cell lines. TGFβ suppresses normal epithelial cell growth but promotes aggressive carcinoma invasiveness and metastasis by inducing EMT [34]. One of the important pathways that change embryonic epithelial cells to mesenchymal cells with enhanced migratory and differentiation capacity is TGFβ signaling. In the kidney TGFβ signaling is involved in the morphogenesis of the mesenchyme at earliest stages of nephrogenesis [35]. Smad4 is the integral component of the signal transduction cascade downstream of ligand receptor activation. Phosphorylation of receptor associated Smads (R-Smads) enhances their affinity for Smad4 and the complex accumulates in the nucleus, where it regulates transcription (activation and repression) [36]. A high expression of *SMAD2* and *SMAD3* is observed in the Wilms cell lines. Furthermore, a large number of TGFβ target genes are expressed in the cells, confirming the activity of this signaling cascade (Figure S13). 

Phosphorylation of CREB and GSKα/β was also found using proteome arrays. The phosphorylation of GSK α/β shows that this kinase is active and can phosphorylate target proteins, with the major target being β-Catenin. Activation of the Wnt signaling pathway in Wilms1 and 2 cell lines was described previously [11]. Here we show that in contrast to the original Wilms3 cell line with wild type *CTNNB1* and no Wnt activity, the Wilms3 cell culture with a mutant *CTNNB1* had an active Wnt signaling pathway. Furthermore, phosphorylation of S15 and S392 in p53 was described to be increased in tumor cells [37] and this was observed in the Wilms cells. 

Signaling via several tyrosine kinases was shown to be deregulated in lung cancers [38]. These can function in independent pathways or participate together in hierarchical networks. In this work we describe that the Wilms tumor cell lines have multiple activated signaling pathways. Using the here described cell culture models of *WT1* mutant Wilms tumors it can now be tested which of the receptors are crucial/essential for cell growth and whether targeting can result in growth arrest or apoptosis. Once the most important signaling pathway has been identified, targeted therapies can be devised. It seems likely that an efficacious therapy for this subtype of *WT1* mutant Wilms tumor might need a combination of drugs targeting these receptors or downstream signaling cascades. The combination of Erlotinib and Imatinib might be a good candidate. Imatinib is already in use for treatment of various PDGFR dependent adult cancers and has little or no side effects. As the cells also have an intrinsic property for differentiation, new approaches to induce differentiation with drugs might be another approach, and could be tested using these established cell lines. 

This subtype of *WT1* mutant Wilms tumor corresponds to cells from an early embryonal stage of kidney precursors that still have the property to migrate. The expression of genes from various lineages in these cells further supports their embryonal stage with a multilineage potential that are not entirely committed to one specific fate. Embryonal cells do not need many mutations to become tumor cells as they still have several of the properties that adult tumors have to regain. In agreement with this is the fact that the *WT1* mutant Wilms8 tumor and cell culture has no additional pathogenic mutation except for *CTNNB1*. 

**Figure 7:**
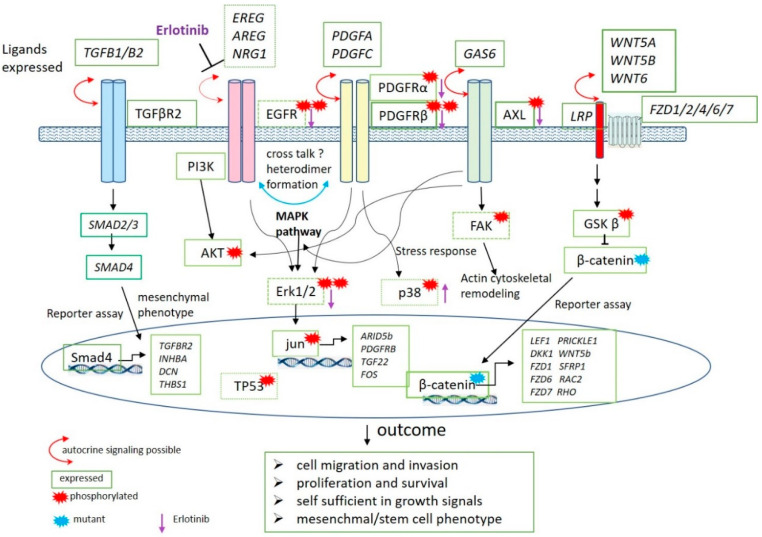
Schematic representation of the data described in this report. The phosphorylated proteins as identified in proteome blots are marked with a red star. Proteins are not in italics and green boxes around the protein indicate the level of the respective RNA expression. The boxes have dark green line when the gene shows a high expression, lighter green corresponds to a lower expression and dotted boxes a low expression. The expressed genes are shown in italics and boxed in green. Included are the data obtained in reporter gene experiments. The location of the proteins in the cell and their function in the pathways is shown. The activity of proteins in the nucleus are indicated in the oval circle and the putative target genes induced by their activity are shown in italics. The mutant β-catenin protein as found in most of the cell lines is labeled with a blue star. The outcomes deduced from these activated pathways are shown below. A purple arrow shows the results of Erlotinib treatment of the cells. Arrow pointing down, less phosphorylation; arrow pointing up, more phosphorylation.

We have summarized the data from the protein studies in context with gene expression results in a schematic drawing (Figure 7). In the model, only results presented in this paper are shown and other steps in the signaling pathways are omitted. Autocrine signaling is possible for all the receptors shown in the Figure, as ligands are expressed at the RNA level. The biological outcome of these activated pathways is shown and the following are the hallmarks of cancer: (1) Cell migration and invasion, (2) proliferation and survival (3) self-sufficiency in growth signals and (4) mesenchymal/stem cell phenotype. This is the first description of a detailed biological insight in this subtype of Wilms tumors and can lead to a better understanding to develop a targeted therapy. Furthermore, these cells will also be useful to study the developmental origin during kidney development.

## 5. Conclusions

Much remains to be elucidated about the molecular basis of the *WT1* mutant Wilms tumor subtype. An in vitro cell culture model is necessary to further explore the embryonal origin and to develop therapeutic options. The method we describe here shows that cell lines can be reproducibly established from these tumors, even if the patient was previously treated with chemotherapy. The induction of mesenchymal differentiation of these cells in vitro, points to the possibility that new agents can be tested for less toxic treatments of patients *WT1* mutant Wilms tumors. The cells can be manipulated by transfection and transduction with lentiviral vectors. The genetically stable immortalized cells are also helpful if more cells are needed for further biochemical studies. 

The proteome activation status of the cells identified several phosphorylated receptors and downstream signaling. The survival of the cells after inhibition of these receptors with established agents can be tested in the future and this will identify which of these is necessary for maintenance of cell growth.

## Figures and Tables

**Table 1:** Mutation status of WT cell lines and tumors.

	Cell Line	*WT1* Mutation Status in Blood DNA of Patient *	*CTNNB1* Mutation in Tumor	*WT1* Mutation Status in Cell Lines *	LOH	*CTNNB1* Mutation in Cell Lines	Patient Treatment before Surgery and Tumor Sampling
1	Wilms1-2r second tumor, right	germ line *WT1* c.149 C > A, p.S50X	right bulk tumor: p.S45F	homozygous c.149 C > A, p.S50X	11p11-11pter	heterozygous TCT > TTT, p.S45F	1 year without chemotherapy ^§^
2	Wilms1-2l second tumor, left	germ line *WT1* c.149 C > A, p.S50X	left bulk tumor: p.S45C	homozygous c.149 C > A, p.S50X	not analyzed	heterozygous TCT > TGT, p.S45C	1 year without chemotherapy
3	Wilms2	germ line *WT1* c.1084 C > T, p.R362X	microdissected: p.ΔS45, p.S45Y, p.S45F; bulk tumor: p.S45F	homozygous c.149 C > A, p.R362X	11p11-11pter	heterozygous TCT > TAT, p.S45Y	no chemotherapy
4	Wilms3	*WT1* wild type	microdissected p.T41A; bulk tumor: p.T41A	homozygous c.1293-1294insA, p.V432SfsX87	11p11-11pter	wild type, early passage ACC > GCC, p.T41A, cells with mutation are lost during culturing	4 weeks ACTD and VCR
5	Wilms4 WAGR	del 11p13	bulk tumor: p.ΔS45	hemizygous c.1311-1312insC, p.H438PfsX79	no LOH in 11p	heterozygous del TCT, p.ΔS45	no chemotherapy
6	Wilms5	germ line *WT1* c.1168 C > T, p.R390X	wild type ^#^	homozygous c.1296-1299delGC, p.R433PfsX84	LOH 11p11-11pter loss of R390X,	wild type	5 weeks ACTD and VCR
7	Wilms6 bilateral tumor	germ line *WT1* c.1168 C > T, p.R390X	left bulk tumor: homozygous p.ΔS45	homozygous c.1168 C > T, p.R390X	11p11-11pter	homozygous del TCT, p.ΔS45	4 weeks ACTD and VCR, 4 weeks Dox
8	Wilms8 bilateral tumor	germ line *WT1* c.1168 C > T, p.R390X	left bulk tumor: p.S45A	homozygous c.1168 C > T, p.R390X	not analyzed	heterozygous TCT > GCT, p.S45A	>7 weeks
9	Wilms10T, primary tumor	*WT1* wild type	primary bulk tumor: heterozygous ACC > GCC p.T41A	homozygous del *WT1* within del11p13	no LOH in 11p13 UPD in 11p15	homozygous del TCT, p.ΔS45, UPD 3p	no chemotherapy
10	Wilms10M, metastasis	*WT1* wild type	lung nodule: heterozygous p.ΔS45, less wild type (mixed population)	homozygous del *WT1* within del11p13	no LOH in 11p13 UPD in 11p15	homozygous del TCT, p.ΔS45, UPD 3p	>6 months, ACTD, VCR, Dox and radiotherapy
11	Wilms11	*WT1* wild type	bulk tumor: heterozygous p.S45F	homozygous c.901 C > T, p.R301X	not analyzed	4 different cell cultures all wild type	no chemotherapy

^#^ the final pathology report of this sample revealed that it corresponds to an intralobar nephroblastomatosis. * Nomenclature for *WT1* mutations: the position of the mutation is shown for isoform D, with exon5 and KTS starting with the first in frame ATG as position 1 (ENST00000379079). ^§^ patient was treated with preoperative chemotherapy before removal of first tumors, thereafter no treatment, second tumors occurred after one year without treatment.

## Data Availability

All cell lines are available upon request.

## Supplementary Materials

Supplementary PDF file is available with the following https://www.mdpi.com/2072-6694/13/1/60/s1, Figure S1: STR marker profile of the Wilms tumor cell lines and their immortalized derivatives; Figure S2: Growth of Wilms1 cells in different media; Figure S3: Immunofluorescence staining of Wilms1,2,6 and 8 with cytokeratin and vimentin; Figure S4: *H19* expression on the Wilms tumor cell lines; Figure S5: Desmin stained slides. The consecutive slide was used for microdissection; Figure S6: Analysis for *WT1* and *CTNNB1* mutations in microdissected material from Wilms3; Figure S7: Immunofluorescence analysis of muscle differentiation potential of Wilms6 and Wilms8 and cardiomyocyte differentiation of Wilms10; Figure S8: Number of cTNT positive cells after differentiation induction; Figure S9: Immunofluorescence staining of Wilms6 cells with CDC42 and RAC1; Figure S10: F-Actin staining of Wilms6 cells with Phalloidin; Figure S11: SBE reporter assays for an activated canonical TGFβ signaling pathway; Figure S12: Western Blots, original films; Figure S13: Heat map of the expression of TGFβ pathway genes. Table S1: Wilms8 tumor specific alterations_filtered; Table S2: Tumor cell culture specific alterations_filtered.
