# Supplementary material for: Comprehensive Biology and Genetics Compendium of Wilms Tumor Cell Lines with Different WT1 Mutations
Source: Cancers (Basel). 2020 Dec 28;13(1):60. doi: 10.3390/cancers13010060 (PMC7801943; doi:10.3390/cancers13010060)
Supplement: Supplementary file 1 [file cancers-13-00060-s001.zip › cancers-1009718 supplementary final/cancers-1009718 supplementary final.pdf]

Supplementary Materials:

Comprehensive Biology and Genetics Compendium of Wilms Tumor Cell Lines with Different *WT1* Mutations

Brigitte Royer-Pokora, Maike Anna Busch, Sarah Tenbusch, Mathias Schmidt, Manfred Beier, Andrew D. Woods, Holger Thiele and Jaume Mora

Name	D3S1358	TH01	D21S11	D18S51	Penta_E	D5S818	D13S317	D7S820	D16S539	CSF1PO	Penta_D	AMEL	vWA	D8S1179	TPOX	FGA	STR result
Wilms1	14, 17	9.3, 9.3	30, 31	15, 18	5, 14	12, 13	11, 13	9, 14	11, 14	10, 12	13, 13	X, X	14, 19	12, 14	8, 9	22, 25	No reference profile, No match
Wilms2	15, 15	6, 6	29, 32.2	12, 17	11, 15	11, 11	9, 11	10, 11	9, 9	11, 11	10, 12	X, Y	15, 19	14, 16	8, 11	21, 21	No reference profile, No match
Wilms3	15, 16	6, 6	29, 31	13, 17	7, 10	9, 9	12, 13	10, 11	9, 11	11, 12	9, 13	X, Y	16, 17	10, 11	8, 8	22, 24	No reference profile, No match
Wilms4	16, 17	7, 8	27, 31.2	16, 17	11, 15	10, 12	8, 8	10, 11	9, 12	10, 11	10, 12	X, Y	15, 17	13, 13	8, 11	20, 20	No reference profile, No match
Wilms5	15, 16	8, 8	28, 29	12, 16	7, 15	10, 13	11, 11	10, 10	8, 12	10, 11	11, 13	X, Y	17, 18	13, 14	9, 10	20, 22	No reference profile, No match
Wilms6	15, 16	9, 9	28, 30	12, 16	8, 13	8, 9	8, 11	8, 11	9, 11	11, 12	11, 11	X, Y	16, 18	11, 13	11, 11	22, 23	No reference profile, No match
Wilms8	16, 18	8, 8	29, 33.2	12, 12	12, 17	12, 13	8, 9	8, 10	13, 13	11, 12	10, 12	X, Y	18, 18	8, 13	8, 9	20, 21	No reference profile, No match
Wilms10M	17, 17	8, 8	29, 30	14, 16	7, 10	10, 12	12, 12	11, 12	9, 10	11, 12	10, 13	X, X	15, 18	10, 15	8, 11	22, 24	No reference profile, No match
Wilms10T	17, 17	8, 8	29, 30	14, 16	7, 10	10, 12	12, 12	11, 12	9, 10	11, 12	10, 13	X, X	15, 18	10, 15	8, 11	22, 24	No reference profile, No match
Wilms11	15, 17	6, 6	29, 30	14, 21	5, 7	12, 13	12, 12	8, 9	11, 12	10, 12	11, 11	X, Y	17, 18	13, 13	9, 11	23, 26	No reference profile, No match
IM-WT-1	14, 17	9.3, 9.3	30, 31	15, 18	5, 14	12, 13	11, 13	9, 14	11, 14	10, 10	13, 13	X, X	14, 19	12, 14	8, 9	22, 25	No reference profile, No match
IM-WT-6	15, 16	9, 9	28, 30	12, 16	8, 13	8, 9	8, 11	8, 11	9, 11	11, 12	11, 11	X, Y	16, 18	11, 13	11, 11	22, 23	No reference profile, No match
IM-WT-10	17, 17	8, 8	29, 30	14, 16	7, 10	10, 12	12, 12	11, 12	9, 10	11, 12	10, 13	X, X	15, 18	10, 15	8, 11	22, 24	No reference profile, No match

Figure S1. STR marker profile for all Wilms tumor cell lines.

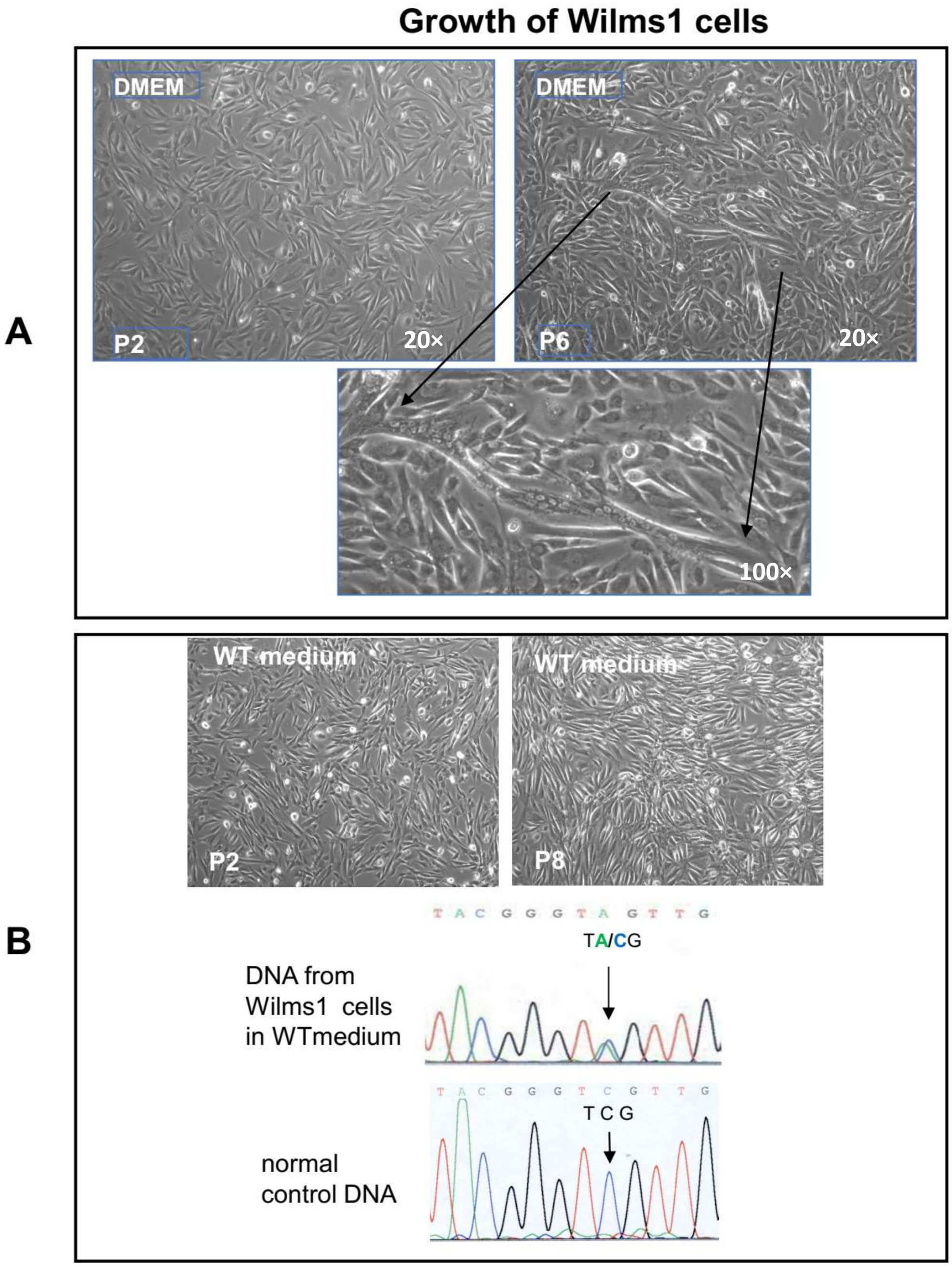

Figure S2. Analysis of Wilms1 cells grown in different media. **A.** Top panel: growth in DMEM and differentiation into multinucleated muscle cells. **B.** Bottom panel: growth in WT medium at passage 2 and 8 and analysis of *WT1* sequence isolated from the cells grown in WT medium. The heterozygous *WT1* mutation in exon1 (p.S50X) is seen. DNA sequence from a normal control person is shown below.

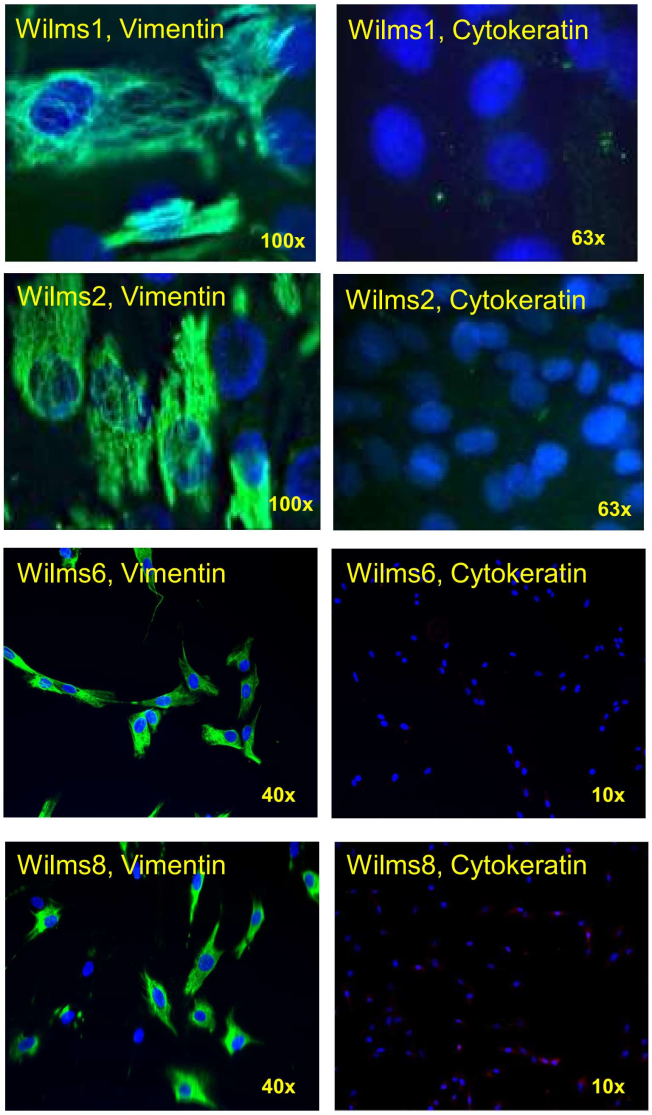

Figure S3. Immunofluorescence staining of cells with Vimentin and cytokeratin. The pictures were captured at different magnifications as indicated.

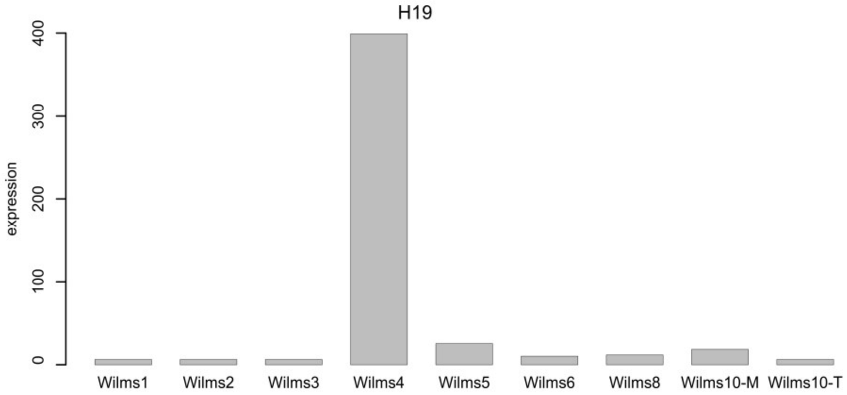

Figure S4. Expression level of H19 in the different cell lines. The level is shown as intensity in Agilent microarrays. All arrays were performed in at least duplicates; for Wilms1, 2,3 and 5 two biological replicates and two experimental replicates were perfomed.

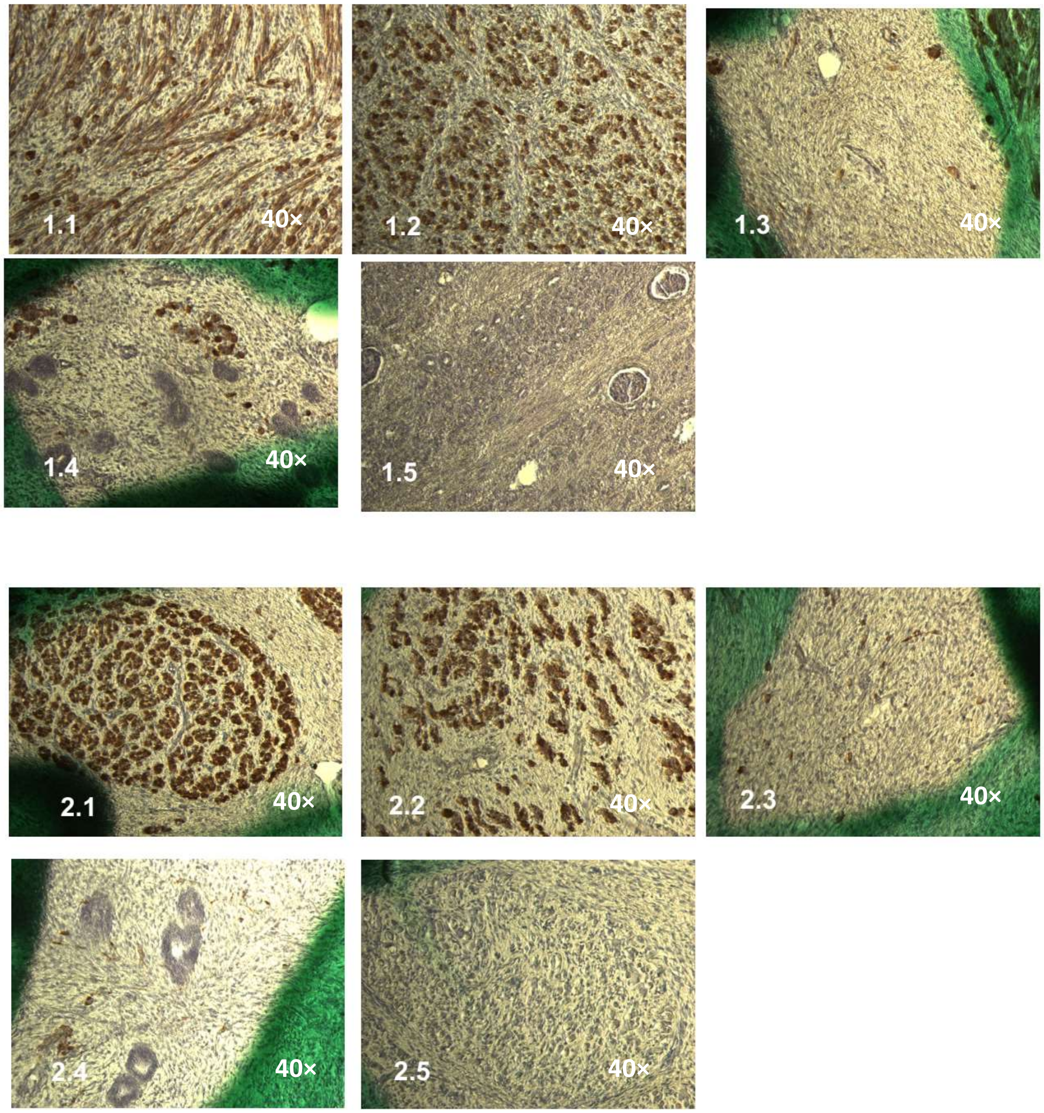

Figure S5. microdissected areas from two different Wilms3 tumor slides. Slide1, 1.1 and 1.2 muscle; 1.3: stroma; 1.4 tubuli; 1.5 normal control kidney Scheme 2. 2.1: muscle; 2.2 muscle; 2.3 stroma; 2.4 tubuli; 2.5 blastema.

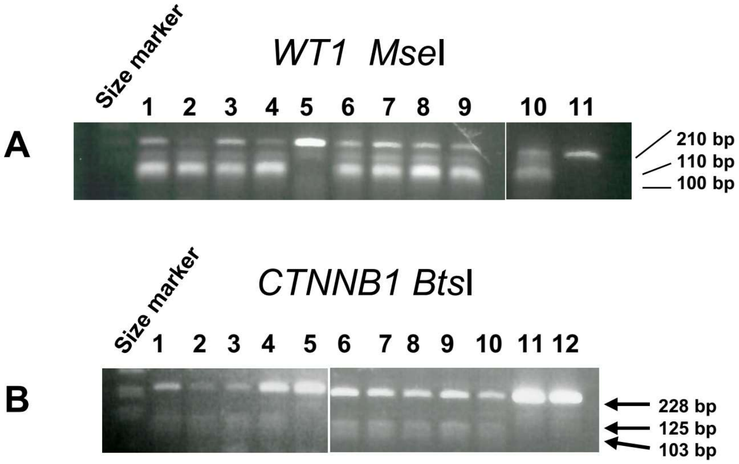

Figure S6. Restriction enzyme digest of PCR products to detect the respective mutations. (**A**) Digestion of the 210 bp *WT1* exon10 PCR product with *Mse*I, the enzyme cuts the mutant PCR product into 110 and 100 bp size fragments (**B**) digestion

of the 228 bp *CTNNB1* PCR product with *Bts*I, this enzyme cuts the mutant PCR product into 125 and 103 bp fragments. Top panel shows a lighter exposure to detect the faint bands of the digested products in lanes 1, 2 3 and 4. Lanes 1: slide 1.1; 2: slide 1.2; 3: slide 1.3; 4: slide.1.4; 5: slide 1.5 normal control area; 6 slide 2.1; 7: slide 2.2; 8: slide 2.3; 9: slide 2.4; 10: slide .2.5; 11: control DNA; 12: control DNA.

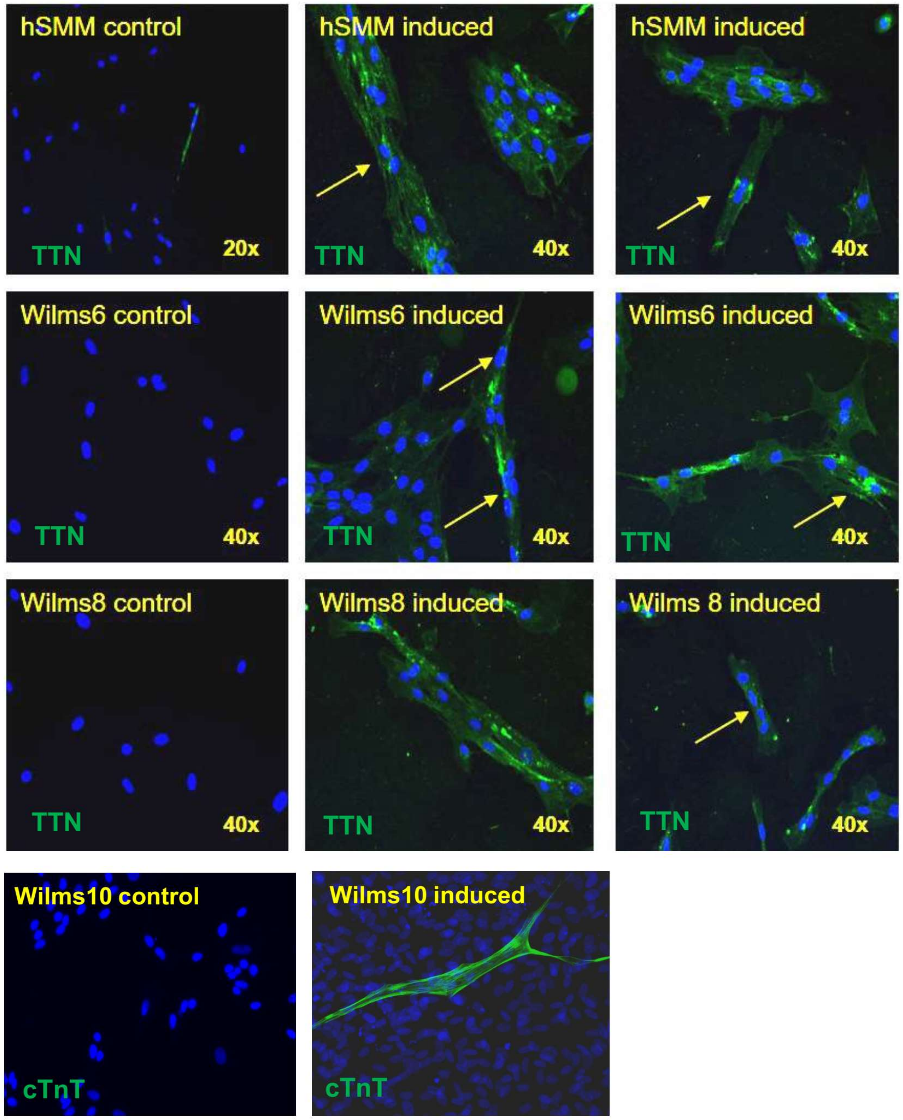

Figure S7. Staining of cells induced for muscle differentiation. As control human skeletal muscle cells (hSMM) were induced at the same time as Wilms 6 and Wilms8 cells. These cells were stained with a Titin antibody (TTN). Muscle induction in Wilms10 cells stained with cardiac troponin T antibody (cTnT).

	Control in growth medium<br>positive cells per view field	growth in serum reduced medium<br>positive cells per view field
cTnT Wilms10	9	140
cTnT Wilms11	7, 6<br>5 cells/100 are positive (4.5%)	59, 70<br>25 cells/87 are positive (29%)

Figure 8. Number of cTnT positive cells after a two week induction of muscle differentiation.

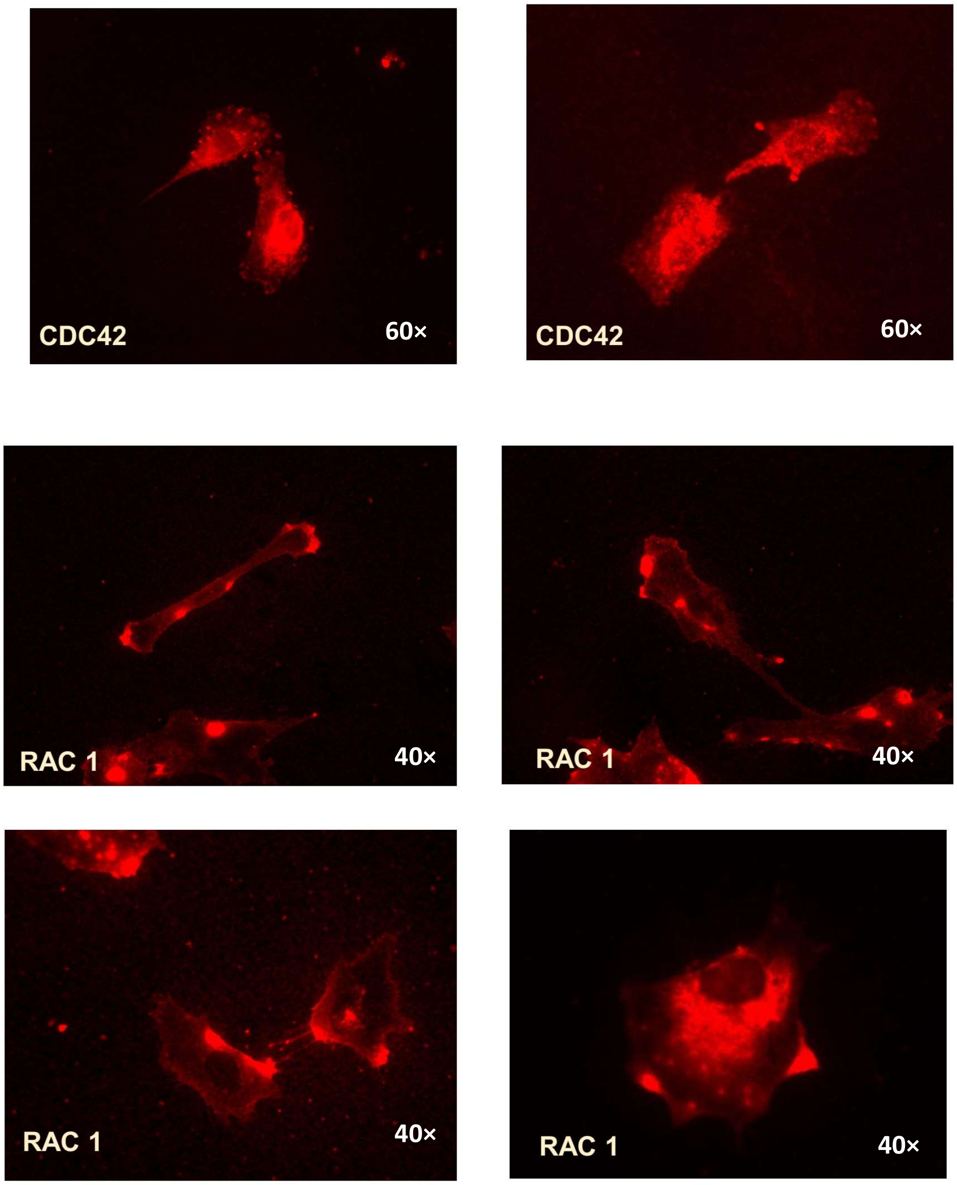

Figure S9. Staining of Wilms6 cells with CDC42 and RAC1.

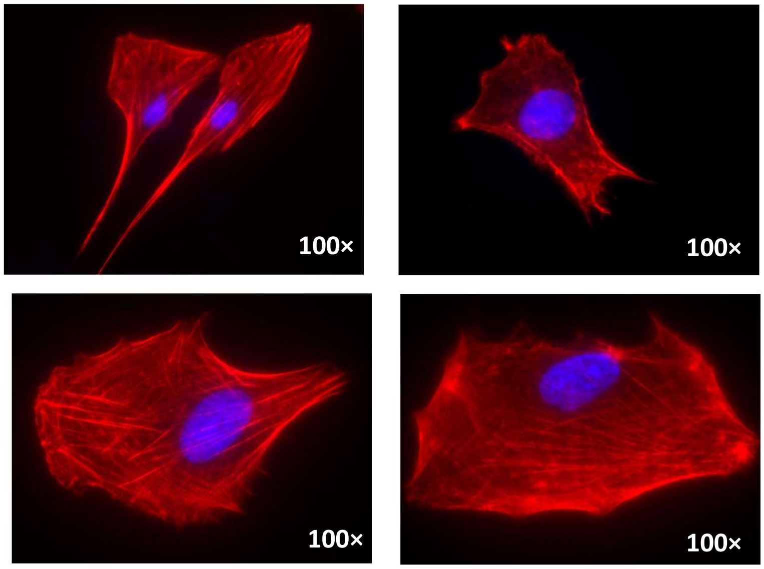

Figure S10. Staining of F-Actin in Wilms6 cells with Rhodamine Phalloidin.

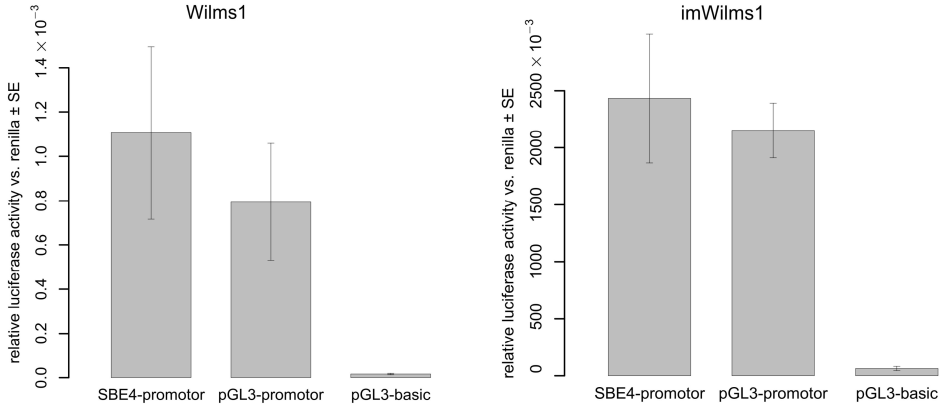

Figure S11. Transfection of SMAD target reporter construct SBE4 into Wilms1 and imWilms1 cells. Transient transfection assay of the Wilms1 (left) and imWilms1 (right) cells with the SBE4 reporter plasmids and the positive control pGL3-promotor and negative pGL3-basic plasmids. SE is shown from three independent transfections.

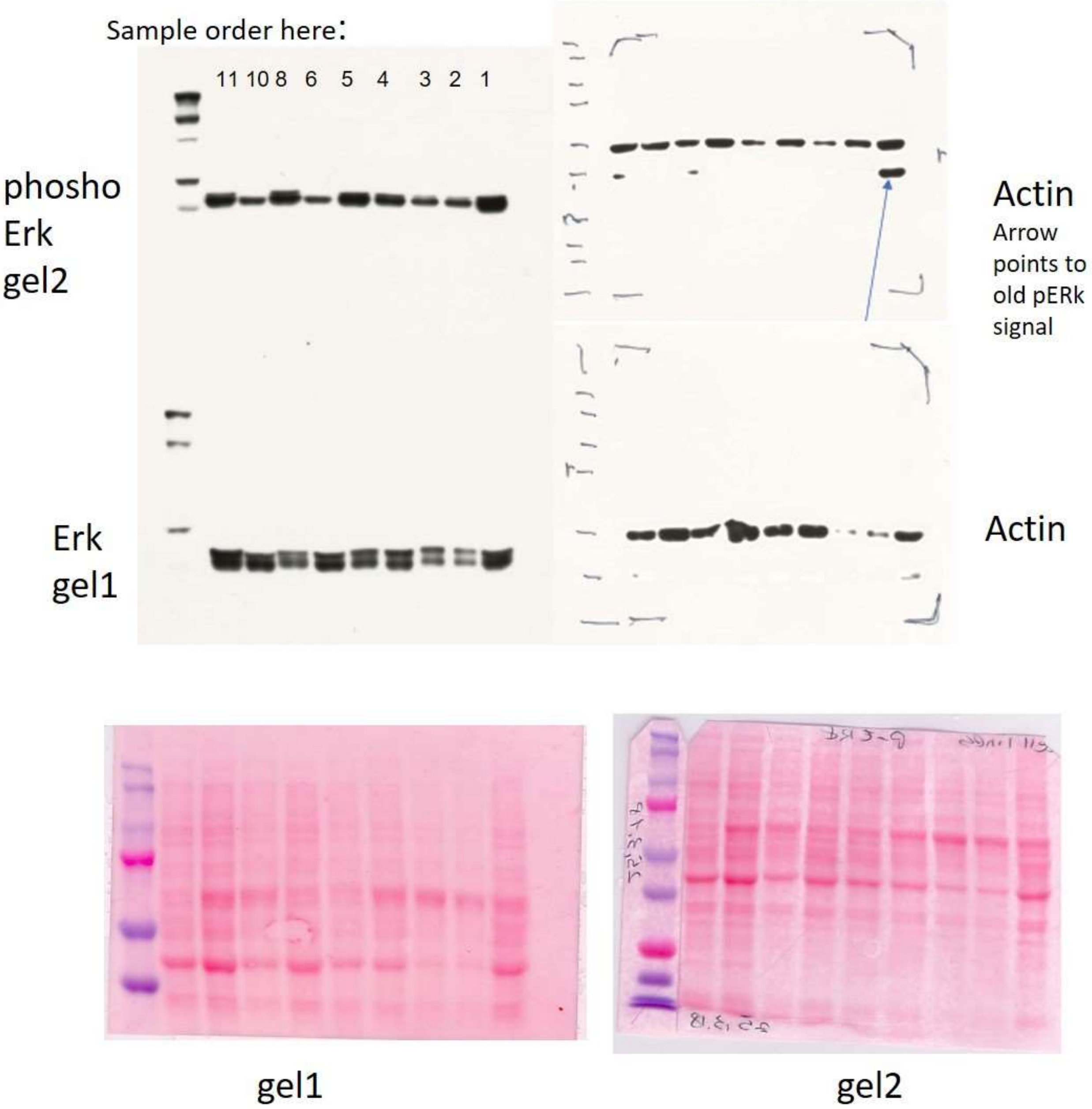

Figure S12. Western blot, original film scans. Top panel shows the original film scans of the blots probed with Erk and phosphoErk antibodies. After removal of bound antibody filters were reprobed with actin antibodies. The corresponding ponceau stained filters are shown below. The order shown here is reverse to the one in the paper, where Wilms1 is on the left and Wilms11 on the right.

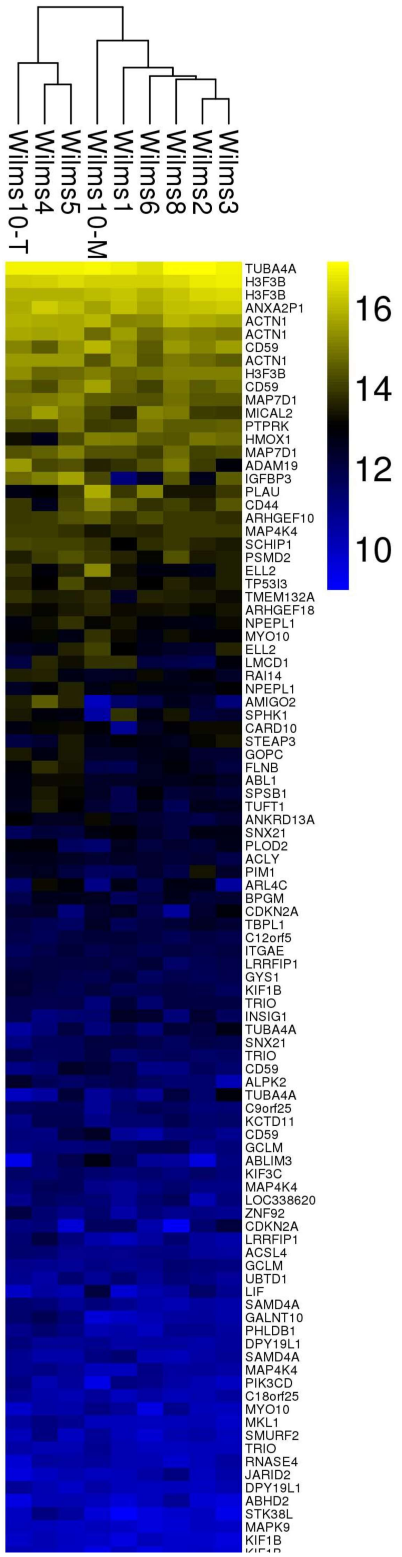

Figure 13. Heat map of the expression of the top TGFRβ induced genes in all Wilms cells. Only genes that are expressed in all of the cell lines above an intensity of 500 are shown
